# An Improved *F_st_* Estimator

**DOI:** 10.1371/journal.pone.0135368

**Published:** 2015-08-28

**Authors:** Guanjie Chen, Ao Yuan, Daniel Shriner, Fasil Tekola-Ayele, Jie Zhou, Amy R. Bentley, Yanxun Zhou, Chuntao Wang, Melanie J. Newport, Adebowale Adeyemo, Charles N. Rotimi

**Affiliations:** 1 Center for Research on Genomics and Global Health, NHGRI, NIH, Bethesda, Maryland, United States of America; 2 Department of Biostatistics, Bioinformatics and Biomathematics, Georgetown University, Washington, DC, United States of America; 3 Suizhou Central Hospital, Suizhou, Hubei, China; 4 Brighton and Sussex Medical School, Falmer, Brighton, United Kingdom; Universitat Pompeu Fabra, SPAIN

## Abstract

The fixation index *F*_*st*_ plays a central role in ecological and evolutionary genetic studies. The estimators of Wright (${\hat{F}}_{st_{1}}$), Weir and Cockerham (${\hat{F}}_{st_{2}}$), and Hudson *et al*. (${\hat{F}}_{st_{3}}$) are widely used to measure genetic differences among different populations, but all have limitations. We propose a minimum variance estimator ${\hat{F}}_{st_{m}}$ using ${\hat{F}}_{st_{1}}$ and ${\hat{F}}_{st_{2}}$. We tested ${\hat{F}}_{st_{m}}$ in simulations and applied it to 120 unrelated East African individuals from Ethiopia and 11 subpopulations in HapMap 3 with 464,642 SNPs. Our simulation study showed that ${\hat{F}}_{st_{m}}$ has smaller bias than ${\hat{F}}_{st_{2}}$ for small sample sizes and smaller bias than ${\hat{F}}_{st_{1}}$ for large sample sizes. Also, ${\hat{F}}_{st_{m}}$ has smaller variance than ${\hat{F}}_{st_{2}}$ for small *F*_*st*_ values and smaller variance than ${\hat{F}}_{st_{1}}$ for large *F*_*st*_ values. We demonstrated that approximately 30 subpopulations and 30 individuals per subpopulation are required in order to accurately estimate *F*_*st*_.

## Introduction

The fixation index *F*_*st*_ is widely used as a measure of population differentiation due to genetic structure. Wright [1, 2] defined *F*_*st*_ as the ratio of the observed variance of allele frequencies between subpopulations to the expected variance of allele frequencies assuming panmixis. Wright’s estimator of *F*_*st*_ is biased, because *a priori* expected allele frequencies are unknown and the numerator and denominator terms in the equation are not independent. In practice, various frameworks have been proposed to improve estimation of *F*_*st*_. Weir and Cockerham used an analysis of variance (ANOVA) approach to estimate within- and between-population variance components [3, 4]. Weir and Cockerham’s estimator is widely used because their estimator can describe the genetic population structure in a single summary statistic, is asymptotically unbiased with respect to sample size, and can compensate for overestimates particularly at low levels of genetic differentiation unlike Wright’s estimator [5]. However, it can be upwardly biased unless adjustment is done for intralocus sampling error, the number of subpopulations sampled, time of divergence, *etc*. [6]. In the present study, we propose a method that improves *F*_*st*_ estimation by combining Wright’s and Weir and Cockerham’s estimators to achieve a minimum variance estimate. For comparison, we also include Hudson et al.’s estimator [7], which recently has been recommended by Bhatia et al. [8]. We demonstrate application of our modified estimator in analysis of real data.

## Methods

For a diallelic marker, let *p* be the true minor allele frequency in the total population. Let the true subpopulation allele frequencies be *p*_1_, …, *p*_*r*_ in *r* ≥ 2 subpopulations. Let *σ* ^2^ be the true population variance in allele frequencies across subpopulations. Suppose the observed sample frequencies are ${\hat{p}}_{1},\ldots ,{\hat{p}}_{r}$ and the sample sizes are *n*_1_, …, *n*_*r*_. Let $n=\sum_{j=1}^{r}n_{j}$ and $\bar{n}=\sum_{j=1}^{r}n_{j}/r$. Let *ϑ* be the difference in allele frequencies, such that for two subpopulations, $\hat{\vartheta }={\hat{p}}_{1}-{\hat{p}}_{2}$.

Wright’s *F*_*st*_ [2] is defined as

$$F_{st}=\frac{\sigma^{2}}{p(1-p)}$$

and is estimated as

$${\hat{F}}_{st_{1}}=\frac{\sum_{j=1}^{r}{\left({\hat{p}}_{j}-\bar{p}\right)}^{2}}{r\bar{p}\left(1-\bar{p}\right)},$$

with

$$\bar{p}=\sum_{j=1}^{r}{\hat{p}}_{j}/r.$$

For the special case of two subpopulations, Rosenberg et al. [9] showed that by algebraic rearrangement

$$F_{st}=\frac{{\left(p_{1}-p_{2}\right)}^{2}}{\left(p_{1}+p_{2}\right)\left(2-\left(p_{1}+p_{2}\right)\right)}.$$

Thus, *F*_*st*_ is a function of the difference in allele frequencies and is proportional to *ϑ* ^2^.

Weir and Cockerham’s estimator [4], assuming a random union of gametes or equivalently no individual-level inbreeding, is based on

$$\bar{p}=\sum_{j=1}^{r}n_{j}{\hat{p}}_{j}/n,$$

$$S^{2}=\frac{1}{\left(r-1\right)\bar{n}}\sum_{j=1}^{r}n_{j}{\left({\hat{p}}_{j}-\bar{p}\right)}^{2},$$

$$T_{1}=S^{2}-\frac{1}{2\bar{n}-1}\left[\bar{p}\left(1-\bar{p}\right)-\frac{r-1}{r}S^{2}\right],$$

$$n_{c}=\frac{1}{r-1}\;(\sum_{j=1}^{r}n_{j}-\frac{\sum_{j=1}^{r}n_{j}^{2}}{\sum_{j=1}^{r}n_{j}}),$$

and

$$T_{2}=\frac{2n_{c}-1}{2\bar{n}-1}\bar{p}\left(1-\bar{p}\right)+\left[1+\frac{2\left(r-1\right)\left(\bar{n}-n_{c}\right)}{2\bar{n}-1}\right]\frac{S^{2}}{r},$$

yielding

$${\hat{F}}_{st_{2}}=\frac{T_{1}}{T_{2}}.$$

The definition of *F*_*st*_ of Hudson et al. [7] is

$$F_{st_{3}}=1-\frac{H_{W}}{H_{B}},\;\;\;\;H_{W}=\frac{1}{r}\sum_{j=1}^{r}2p_{j}\left(1-p_{j}\right),\;\;\;H_{B}=\frac{1}{r(r-1)}\sum_{i\neq j}^{r}2p_{i}\left(1-p_{j}\right).$$

Given observed sample estimates ${\hat{p}}_{1},\ldots ,{\hat{p}}_{r}$, ${\hat{H}}_{W}=r^{-1}\sum_{j=1}^{r}2{\hat{p}}_{j}(1-{\hat{p}}_{j})$ is a biased estimate of *H*_*W*_, because

$$\begin{array}{c} E\;\left({\hat{p}}_{j}\;\left(1-{\hat{p}}_{j}\right)\right)=E\;\left({\hat{p}}_{j}-{\hat{p}}_{j}^{2}\right)=E\;\left({\hat{p}}_{j}\right)-E\;\left({\hat{p}}_{j}^{2}\right)\\ =p_{j}-\;\left(p_{j}^{2}+\frac{p_{j}\;\left(1-p_{j}\right)}{2n_{j}}\right)=p_{j}\;\left(1-p_{j}\right)\;\left(1-\frac{1}{2n_{j}}\right). \end{array}$$

An unbiased estimate of *p*_*j*_(1 − *p*_*j*_) is thus given by $\left[2n_{j}/(2n_{j}-1)]{\hat{p}}_{j}(1-{\hat{p}}_{j}\right)$. However, ${\hat{H}}_{B}={\left[r(r-1)\right]}^{-1}\sum_{i\neq j}^{r}2{\hat{p}}_{i}(1-{\hat{p}}_{j})$ is an unbiased estimate of *H*_*B*_ if $Cov({\hat{p}}_{i},{\hat{p}}_{j})=0$, i.e., under the null hypothesis. Therefore, we estimate *F*_*st*_ by

$${\hat{F}}_{st_{3}}=1-\frac{\left(r-1\right)\sum_{j=1}^{r}\frac{2n_{j}}{2n_{j}-1}{\hat{p}}_{j}\left(1-{\hat{p}}_{j}\right)}{\sum_{i\neq j}^{r}{\hat{p}}_{i}\left(1-{\hat{p}}_{j}\right)},$$

which is a ratio of unbiased estimates. This estimator generalizes Bhatia et al.’s [8] version of Hudson et al.’s [7] estimator for *r* > 2.

Note that under the null hypothesis of *p*_1_ = ⋯ = *p*_*r*_, both $\bar{n}{\hat{F}}_{st_{1}}$ and $\bar{n}{\hat{F}}_{st_{2}}$ are asymptotically zero. Our goal is to construct an estimator based on a linear combination of $\bar{n}{\hat{F}}_{st_{1}}$ and $\bar{n}{\hat{F}}_{st_{2}}$ such that the new estimator has the smallest variance among all such linear combinations. Let $\sigma_{1}^{2}$ and $\sigma_{2}^{2}$ be the asymptotic variances of $\bar{n}{\hat{F}}_{st_{1}}$ and $\bar{n}{\hat{F}}_{st_{2}}$, and *σ*_12_ be the asymptotic covariance. We propose the following weighted version of ${\hat{F}}_{st}$:

$${\hat{F}}_{st_{m}}={\hat{F}}_{st}(a)=a{\hat{F}}_{st_{1}}+(b-a){\hat{F}}_{st_{2}},a>0,$$

where *b* > 0 is a fixed number to be chosen later. We choose *a* = *a*_0_ such that $Var({\hat{F}}_{st}(a))$ is minimized:

$$a_{0}=\text{arg}\;\underset{a>0}{\text{min}}\left\{a^{2}\sigma_{1}^{2}+{\left(b-a\right)}^{2}\sigma_{2}^{2}+2a(b-a)\sigma_{12}\right\}.$$ (1)

It is seen that $Var({\hat{F}}_{st}(a_{0}))\leq \text{min}\{Var({\hat{F}}_{st_{1}}),Var({\hat{F}}_{st_{2}})\}$ and hence is more precise in estimation. From the proof of the Proposition we see that Eq (1) is equivalent to,

$$a_{0}=\text{arg}\;\underset{a>0}{\text{min}}\left\{a^{2}+{\left(b-a\right)}^{2}\delta^{2}+2a(b-a)\delta \right\},\;\;\delta =\underset{n\to \infty }{\text{lim}}\bar{n}/n_{c}.$$ (2)

which gives, with *b* = (*δ* − 1)/(*δ* + 1),

$$a_{0}=\text{arg}\;\underset{a>0}{\text{min}}\left\{a^{2}{\left(\delta -1\right)}^{2}-2ab\delta (\delta -1)+\delta^{2}\right\}=\frac{\delta }{\delta +1}.$$

At the end of the proof of the following Proposition, we show that *δ* ≥ 1 with equality if and only if *n*_1_ = ⋯ = *n*_*r*_. When *n*_1_ = ⋯ = *n*_*r*_, we have $\bar{n}=n_{c}$ and ${\hat{F}}_{st}(a_{0})=\frac{1}{2}{\hat{F}}_{st_{1}}-\frac{1}{2}{\hat{F}}_{st_{2}}$. Let $\overset{D}{\to }$ denote convergence in distribution.

**Proposition**. *Assume that 0 < p*_0_ < 1 *and that the n_j_’s are not all equal (so that δ > 1). If p_1_ = ⋯ = p_r_, with*

$$\delta =\underset{n\to \infty }{\text{lim}}\bar{n}/n_{c}$$

*and*

$$\gamma_{j}=\underset{n\to \infty }{\text{lim}}n_{j}/n,$$

*we have*

$$\bar{n}{\hat{F}}_{st}(a)+\delta (1-a)\overset{D}{\to }\frac{a+\delta (1-a)}{\left(r-1)p_{0}(1-p_{0}\right)}\sum_{j=1}^{r}\lambda_{j}\chi_{j}^{2},$$

*where λ*_1_, *…,λ_r_ are the eigenvalues of* Ω′^1/2^ **B**Ω^1/2^, Ω = (*ω*_*ij*_)_*r* × *r*_ *with* *ω*_*ij*_ = *p*_0_(1 − *p*_0_) *if* *i* = *j* *and* *ω*_*ij*_ = 0 *if* *i* ≠ *j*, Ω^−1/2^ *is the square root of* Ω: Ω = Ω′^1/2^Ω^1/2^, *and* $\text{B}=\sum_{j=1}^{r}\gamma_{j}{\text{b}}_{j}{\text{b}}^{\prime_{j}}$, **b**_*j*_ = (−*γ*_1_, …, −*γ*_*j*−1_, (1 − *γ*_*j*_), −*γ*_*j*+1_, …, −*γ*_*r*_)′.

In the above Proposition, take *a* = *a*_0_, then *a*_0_+*δ*(1 − *a*_0_) = 0 and *δ* (1 − *a*_0_) = −*δ*/(*δ* − 1), and we get

**Corollary 1**. *Under conditions of the Proposition,*

- $$\bar{n}{\hat{F}}_{st}(a_{0})+\frac{\delta }{\delta +1}\overset{D}{\to }\frac{2\delta }{\left(r-1)(\delta +1)p_{0}(1-p_{0}\right)}\sum_{j=1}^{r}\lambda_{j}\chi_{j}^{2}.$$
- *If *a* = 1, then*
  $$\bar{n}{\hat{F}}_{st_{1}}=\bar{n}{\hat{F}}_{st}(1)\overset{D}{\to }\frac{1}{\left(r-1)p_{0}(1-p_{0}\right)}\sum_{j=1}^{r}\lambda_{j}\chi_{j}^{2}.$$
- *If *a* = 0, then*
  $$\bar{n}{\hat{F}}_{st_{2}}=\bar{n}{\hat{F}}_{st}(0)+\delta \overset{D}{\to }\frac{\delta }{\left(r-1)p_{0}(1-p_{0}\right)}\sum_{j=1}^{r}\lambda_{j}\chi_{j}^{2}.$$

**Simulations** Under the Balding-Nichols model [10], the allele frequency in each of *r* subpopulations conditional on *p* and *F*_*st*_ is a random deviate from the beta distribution *β* $\left(\frac{1-F_{st}}{F_{st}}p,\frac{1-F_{st}}{F_{st}}(1-p)\right)$, which has mean *p* and variance *p*(1 − *p*)*F*_*st*_ = *σ* ^2^.

**Simulation 1**. This simulation was designed to estimate bias in the worst case scenario of two subpopulations. We evaluated the relationships between ${\hat{F}}_{st}$ and *F*_*st*_ and between ${\hat{F}}_{st}$ and $\hat{\vartheta }$. First, given the true average allele frequency *p* for *r* = 2, *F*_*st*_ reaches its maximum value for *p*_*j*_ values of 0 and 2*p*. The estimator ${\hat{F}}_{st}=\frac{S^{2}}{\bar{p}(1-\bar{p})+\frac{S^{2}}{r}}$ [4] yields a constrained range for ${\hat{F}}_{st}$ from 0 to 2*p*. Therefore, we first assigned the true value for *p* by drawing a random uniform deviate from the interval (0, 0.5) and the true value for *F*_*st*_ by independently drawing a random uniform deviate from the interval (0, 2*p*). Conditional on the true values of *p* and *F*_*st*_, we randomly generated *p*_*j*_ from the beta distribution. We next assigned the number of individuals per subpopulation *n*_*j*_ = [5, 10, 20, 50, 100, 110]. We then randomly drew alleles from the binomial distribution *Bin*(2*n*_*j*_, *p*_*j*_). We generated 10,000 independent replicate data sets. Based on the above formulae, the four estimators ${\hat{F}}_{st_{1}}$, ${\hat{F}}_{st_{2}}$, ${\hat{F}}_{st_{3}}$, and ${\hat{F}}_{st_{m}}$ were calculated. Linear regression models were used to evaluate the relationship between *F*_*st*_ and ${\hat{F}}_{st}$ and between ${\hat{F}}_{st}$ and ${\hat{\vartheta }}^{2}$. We assessed the fit in a linear regression model with the *F*-test, *r* ^2^, and the root mean squared error (RMSE), which is the square root of the sum of the variance and the squared bias.

**Simulation 2**. This simulation was designed to evaluate variance under sampling conditions approaching unbiasedness, i.e., large numbers of subpopulations and individuals per subpopulation. We evaluated the relationships between $\hat{F_{st}}$ and the number of subpopulations (*r*) and between $\hat{F_{st}}$ and the number of individuals per subpopulations (*n*_*j*_). Conditional on the average allele frequency *p*, *F*_*st*_, the number of subpopulations *r* = [5, 10, 20, 50, 100, 250], and the number of individuals per subpopulation *n*_*j*_ = [5, 10, 20, 50, 100, 250, 1000], we randomly generated *r* allele frequencies as in Simulation 1 and calculated ${\hat{F}}_{st_{1}}$, ${\hat{F}}_{st_{2}}$, ${\hat{F}}_{st_{m}}$, and ${\hat{F}}_{st_{3}}$.

### Application to data

We included genotype data from a total of 120 unrelated individuals from the Wolaita (WETH) ethnic group from southern Ethiopia who served as controls in a genome-wide association study of podoconiosis [11]. The Wolaita ethnic group speaks an Omotic language, and comparison with HapMap African populations has shown that it has the closest genetic similarity with the Maasai from Kenya and the lowest genetic similarity with the Yoruba in Nigeria [12]. Genotyping was performed by deCODE Genetics using the Illumina HumanHap 610 Bead Chip, which assays > 620,000 single-nucleotide polymorphisms (SNPs). Of the 551,840 autosomal SNPs in the raw genotype data, we excluded 39,249 SNPs that had a minor allele frequency of < 0.05, 378 that were missing in > 0.05 of individuals, and 321 that had a Hardy-Weinberg *p*-value < 0.001. The remaining 511,892 SNPs were merged with genotype data for ASW (*n* = 49), CEU (*n* = 112), CHB (*n* = 84), CHD (*n* = 85), GIH (*n* = 88), JPT (*n* = 86), LWK (*n* = 90), MKK (*n* = 143), MXL (*n* = 50), TSI (*n* = 88), and YRI (*n* = 113) in HapMap phase 3, release 2, which contained 1,440,616 SNPs. A total of 464,642 SNPs were common to both of WETH and HapMap data sets. ${\hat{F}}_{st_{1}}$ ${\hat{F}}_{st_{2}}$, ${\hat{F}}_{st_{m}}$, and ${\hat{F}}_{st_{3}}$ were calculated per marker.

## Results

**Fig 1:**
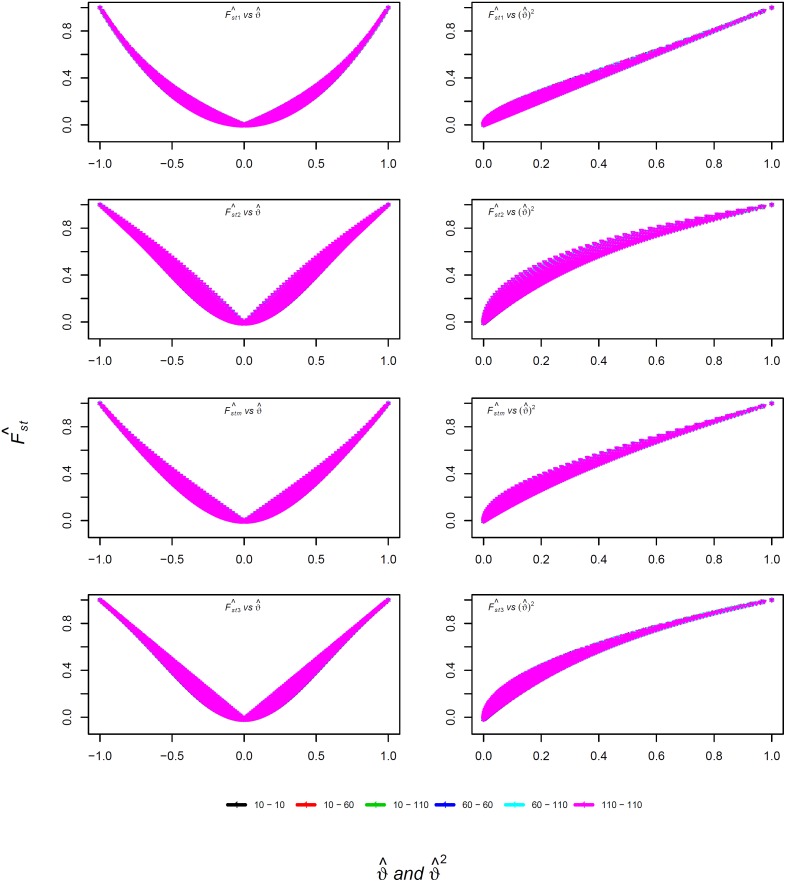
The relationship between ${\hat{F}}_{st}$ and $\hat{\vartheta }$ for simulated data. The x-axis shows the difference of allele frequencies between two subpopulations $\hat{\vartheta }$ (left plots) and ${\hat{\vartheta }}^{2}$ (right plots); the y-axis shows ${\hat{F}}_{st}$ values for Wright’s (top row), Weir and Cockerham’s (second row), the modified (third row), and Hudson et al.’s estimators (bottom row), and the legend indicates the sample sizes *n*_1_ (before hyphen) and *n*_2_ (after hyphen).

Simulation 1: We first compared ${\hat{F}}_{st}$ with the true *F*_*st*_ for the worst-case scenario of *r* = 2. For small sample sizes, ${\hat{F}}_{st_{1}}$ was the least biased estimator, followed by ${\hat{F}}_{st_{m}}$, ${\hat{F}}_{st_{2}}$, and ${\hat{F}}_{st_{3}}$ (Table 1). For large sample sizes, ${\hat{F}}_{st_{m}}$ and ${\hat{F}}_{st_{1}}$ were comparably good, and ${\hat{F}}_{st_{2}}$ and ${\hat{F}}_{st_{3}}$ were identically worse (Table 1). None of the four estimators was strongly sensitive to equal vs. unequal sample sizes (Fig 1). When $\hat{\vartheta }$ was close to 0, ${\hat{F}}_{st_{3}}$ yielded the most negative estimates, followed by ${\hat{F}}_{st_{2}}$ and ${\hat{F}}_{st_{m}}$. As expected, all four estimators showed a quadratic relationship with $\hat{\vartheta }$ (Fig 1). With respect to ${\hat{\vartheta }}^{2}$, by all four measures ${\hat{F}}_{st_{1}}$ was the best estimator whereas ${\hat{F}}_{st_{2}}$ was the worst estimator (Table 1).

**Table 1:** ${\hat{F}}_{st}$ vs. *F*_*st*_ and ${\hat{\vartheta }}^{2}$ for two subpopulations.

	${\hat{F}}_{st_{1}}$	${\hat{F}}_{st_{2}}$	${\hat{F}}_{st_{m}}$	${\hat{F}}_{st_{3}}$
*n* = 5				
${\hat{F}}_{st}$ vs. *F*_*st*_				
Root MSE	0.2213	0.2241	0.2228	0.2241
Squared bias	0.0465	0.0481	0.0473	0.0484
*r* ^2^	0.1096	0.0869	0.0979	0.0869
*F*-test	12310	9522	10848	9522
${\hat{F}}_{st}$ vs. $\hat{\vartheta^{2}}$				
Root MSE	0.0340	0.1002	0.0657	0.1127
Squared bias	0.0008	0.0089	0.0036	0.0114
*r* ^2^	0.9855	0.9129	0.9549	0.9129
*F*-test	6.808 × 10^6^	1.048 × 10^6^	2.116 × 10^6^	1.048 × 10^6^
*n* = 1000				
${\hat{F}}_{st}$ vs. *F*_*st*_				
Root MSE	0.2102	0.2105	0.2101	0.2105
Squared bias	0.0415	0.0419	0.0416	0.0419
*r* ^2^	0.1981	0.1958	0.1986	0.1958
*F*-test	24701	24344	24778	24344
${\hat{F}}_{st}$ vs. $\hat{\vartheta^{2}}$				
Root MSE	0.0233	0.0706	0.0455	0.0706
Squared bias	0.0002	0.0041	0.0015	0.0041
*r* ^2^	0.9913	0.9355	0.9700	0.9355
*F*-test	1.140 × 10^7^	1.450 × 10^6^	3.236 × 10^6^	1.450 × 10^6^

**Fig 2:**
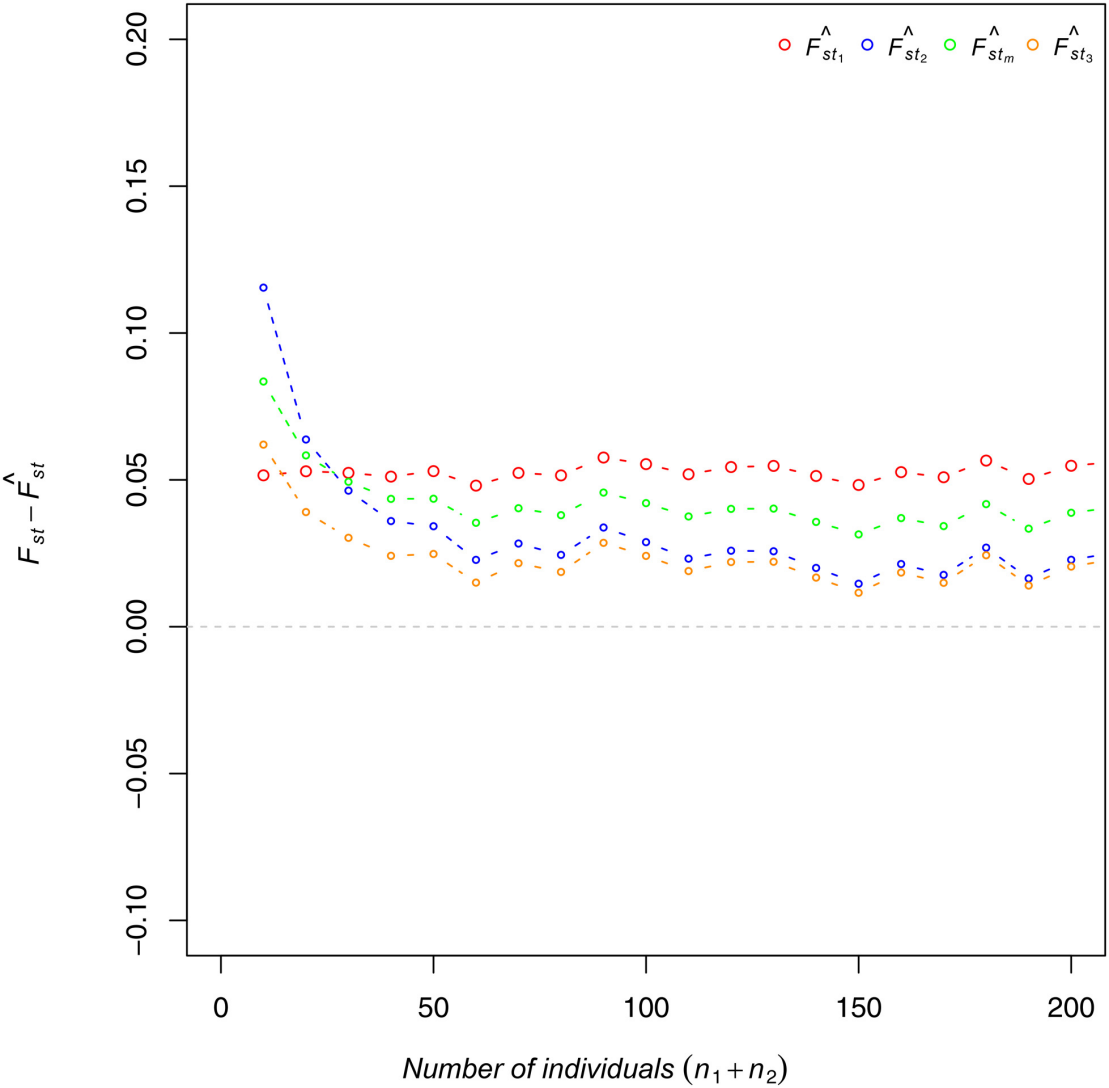
Bias as a function of total sample size. The x-axis shows the total sample size (*n*_1_ + *n*_2_). The y-axis shows $F_{st}-{\hat{F}}_{st_{1}}$ (red), $F_{st}-{\hat{F}}_{st_{2}}$ (blue), $F_{st}-{\hat{F}}_{st_{m}}$ (green), and $F_{st}-{\hat{F}}_{st_{3}}$ (orange) for *r* = 2.

An assessment of bias by the total sample size (*n*_1_ and *n*_2_) for *r* = 2 is presented in Fig 2. ${\hat{F}}_{st_{1}}$ was biased and this bias was constant across total sample size, as expected given that this estimator does not account for *n*_*j*_. In contrast, ${\hat{F}}_{st_{2}}$, ${\hat{F}}_{st_{m}}$, and ${\hat{F}}_{st_{3}}$ were less biased as the total sample size increased. When the total sample size exceeded 30, ${\hat{F}}_{st_{3}}$ was the least biased estimator; otherwise, ${\hat{F}}_{st_{1}}$ was the least biased estimator. For *r* = 2, the magnitude of bias for all four estimators was constant when the total sample size was at least 60.

**Fig 3:**
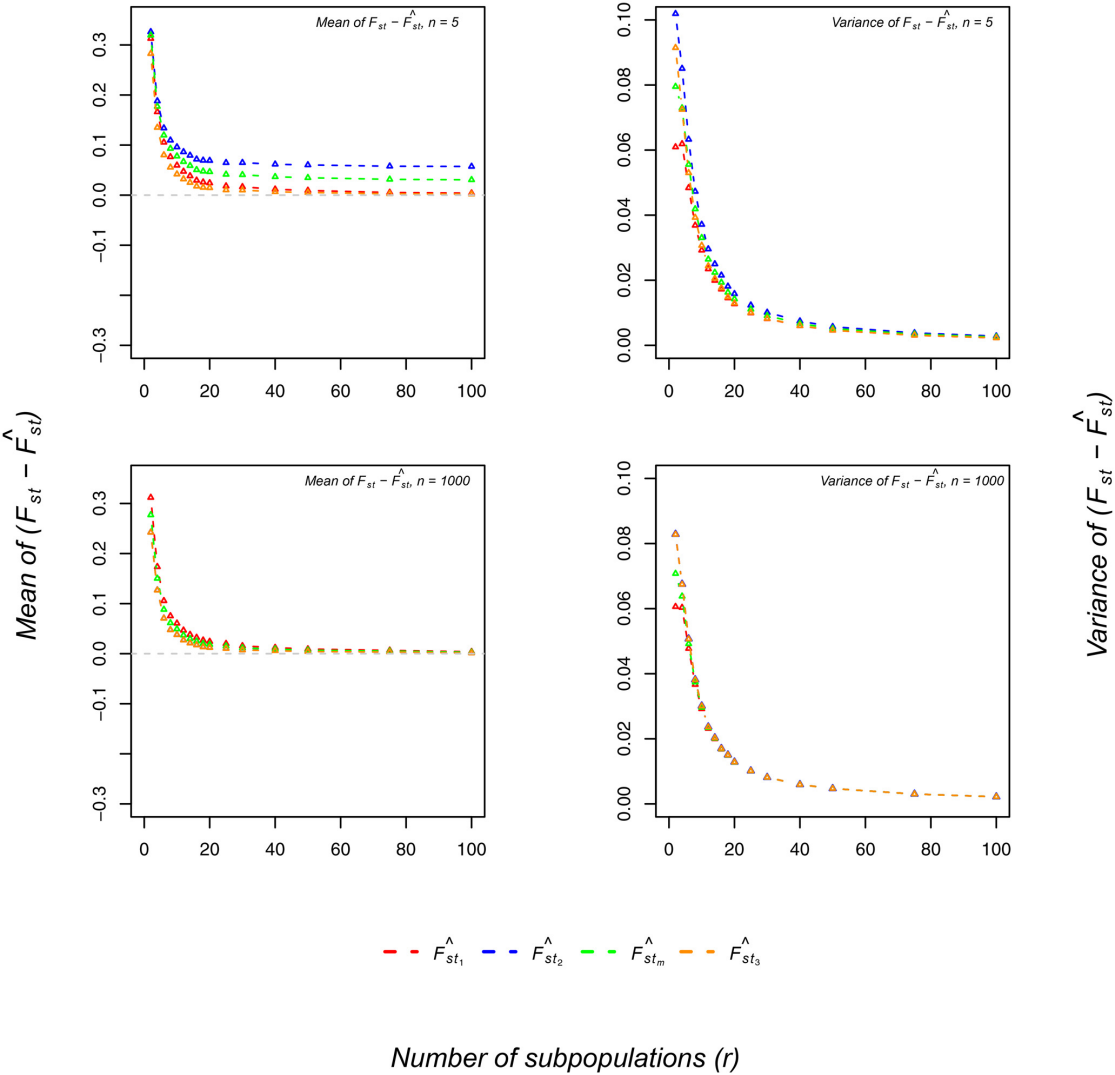
Effect of the number of subpopulations on bias. The x-axis shows the number of subpopulations. The y-axis shows the mean (left) and variance (right) of $F_{st}-{\hat{F}}_{st_{1}}$ (red), $F_{st}-{\hat{F}}_{st_{2}}$ (blue), $F_{st}-{\hat{F}}_{st_{m}}$ (green), and $F_{st}-{\hat{F}}_{st_{3}}$ (orange) values, given *F*_*st*_ = 0.5 and average allele frequency *p* = 0.2. The top plot represents 5 individuals per subpopulation and the bottom plot represents 1000 individuals per subpopulation.

Simulation 2: Given *p* = 0.2, *F*_*st*_ = [0.1, 0.2, 0.3, 0.4, 0.5, 0.6, 0.7, 0.8, 0.9], *n* = 1000 individuals, and *r* = 200 subpopulations, mean ${\hat{F}}_{st}$ values are presented in Table 2. The means for ${\hat{F}}_{st_{1}}$, ${\hat{F}}_{st_{2}}$, ${\hat{F}}_{st_{3}}$, and ${\hat{F}}_{st_{m}}$ equaled the expected values, consistent with all four estimators being asymptotically unbiased. First, we investigated the relationship between *F*_*st*_ and the variance of the four estimators. Given *p* = 0.2 and *F*_*st*_ < 0.5, ${\hat{F}}_{st_{1}}$ had the smallest variance, followed by ${\hat{F}}_{st_{m}}$ and ${\hat{F}}_{st_{2}}$ (Fig 3). Given *p* = 0.2 and *F*_*st*_ > 0.5, ${\hat{F}}_{st_{2}}$ had the smallest variance, followed by ${\hat{F}}_{st_{m}}$ and ${\hat{F}}_{st_{1}}$. Similar results were obtained for *p* = 0.1, 0.3, 0.4, and 0.5 (S1 Table).

**Table 2:** Means, Variances, and MSEs of ${\hat{F}}_{st}$ in simulation 2.

True *F*_*st*_		${\hat{F}}_{st_{1}}^{*}$	${\hat{F}}_{st_{2}}$	${\hat{F}}_{st_{m}}$	${\hat{F}}_{st_{3}}$
0.1	Means	9.95E-02	9.95E-02	9.95E-02	9.99E-02
	Variances	9.40E-05	9.49E-05	9.44E-05	9.48E-05
	MSE	9.43E-05	9.51E-05	9.47E-05	9.48E-05
0.2	Means	1.99E-01	1.99E-01	1.99E-01	2.00E-01
	Variances	3.36E-04	3.38E-04	3.37E-04	3.38E-04
	MSE	3.37E-04	3.39E-04	3.38E-04	3.38E-04
0.3	Means	2.99E-01	2.99E-01	2.99E-01	3.00E-01
	Variances	6.27E-04	6.30E-04	6.28E-04	6.29E-04
	MSE	6.29E-04	6.30E-04	6.29E-04	6.29E-04
0.4	Means	3.98E-01	3.99E-01	3.99E-01	3.99E-01
	Variances	9.12E-04	9.15E-04	9.14E-04	9.14E-04
	MSE	9.15E-04	9.16E-04	9.15E-04	9.14E-04
0.5	Means	4.98E-01	4.99E-01	4.98E-01	4.99E-01
	Variances	1.11E-03	1.11E-03	1.11E-03	1.11E-03
	MSE	1.11E-03	1.11E-03	1.11E-03	1.11E-03
0.6	Means	5.98E-01	5.99E-01	5.99E-01	6.00E-01
	Variances	1.18E-03	1.18E-03	1.18E-03	1.18E-03
	MSE	1.18E-03	1.18E-03	1.18E-03	1.18E-03
0.7	Means	6.98E-01	6.99E-01	6.99E-01	6.99E-01
	Variances	1.12E-03	1.12E-03	1.12E-03	1.11E-03
	MSE	1.12E-03	1.12E-03	1.12E-03	1.11E-03
0.8	Means	7.98E-01	7.99E-01	7.99E-01	7.99E-01
	Variances	8.67E-04	8.63E-04	8.65E-04	8.62E-04
	MSE	8.69E-04	8.64E-04	8.66E-04	8.63E-04
0.9	Means	8.99E-01	8.99E-01	8.99E-01	8.99E-01
	Variances	4.81E-04	4.77E-04	4.79E-04	4.77E-04
	MSE	4.81E-04	4.78E-04	4.79E-04	4.77E-04

* Means, variances, and Mean Squared Errors (MSEs) from 200 subpopulations with 1000 individuals per subpopulation, given *p* = 0.2.

**Fig 4:**
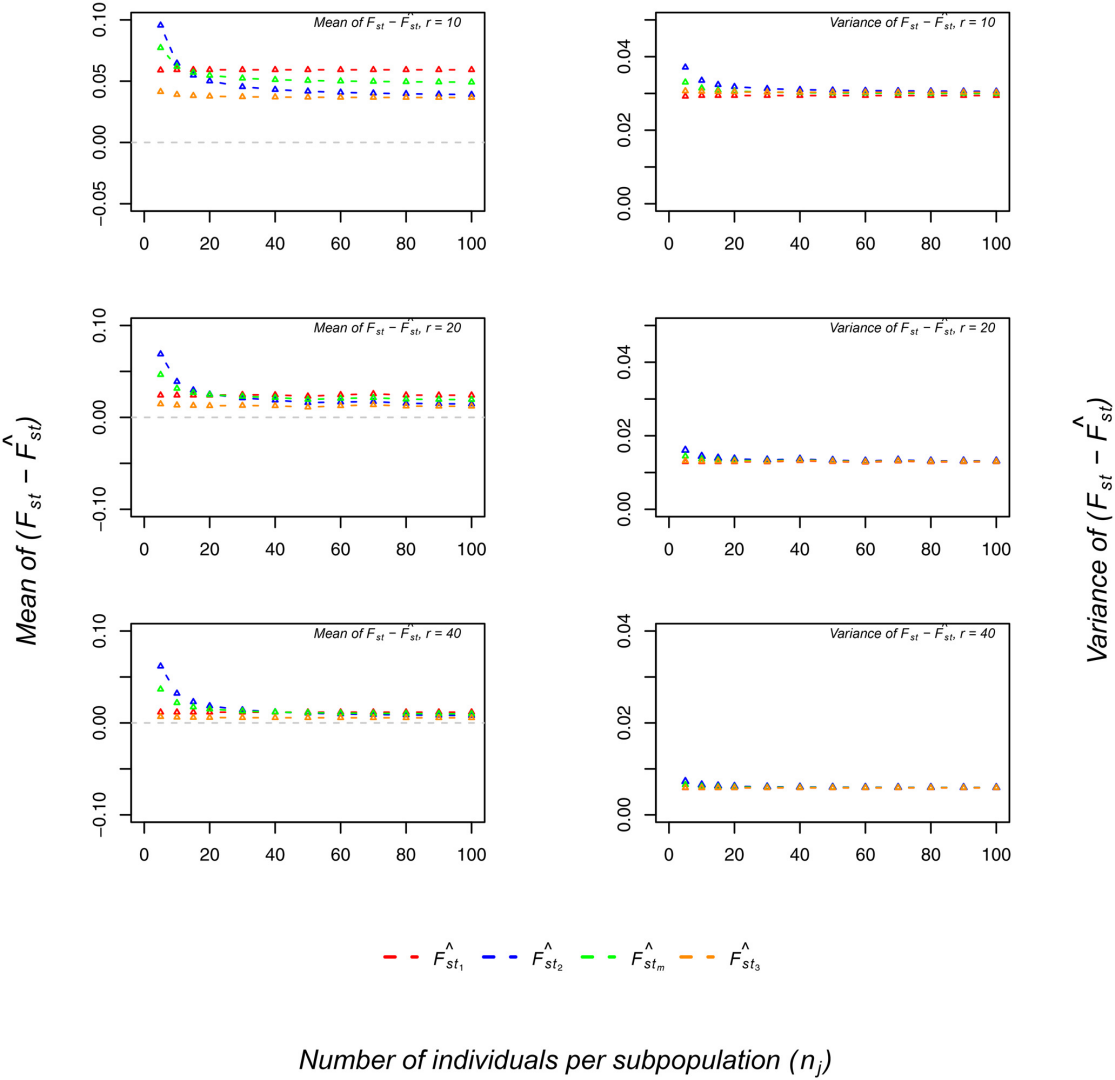
Effect of the number of individuals per subpopulation on bias. The x-axis shows the number of individuals per subpopulation. The y-axis shows the mean (left) and variance (right) of $F_{st}-{\hat{F}}_{st_{1}}$ (red), $F_{st}-{\hat{F}}_{st_{2}}$ (blue), $F_{st}-{\hat{F}}_{st_{m}}$ (green), and $F_{st}-{\hat{F}}_{st_{3}}$ (orange) values, given *F*_*st*_ = 0.5 and an average allele frequency *p* = 0.2. From top to bottom, the plots represent the number of subpopulations *r* = 10, 20, and 40, respectively.

Second, we investigated how the number of subpopulations and the number of individuals per subpopulation affected bias. When the number of subpopulations was approximately 30, no matter the number of individuals per subpopulation, bias was stable (Fig 3). For *r* > 30 and small *n*_*j*_, all four estimators were biased, with the order of ${\hat{F}}_{st_{1}}<{\hat{F}}_{st_{3}}<{\hat{F}}_{st_{m}}<{\hat{F}}_{st_{2}}$. For *r* > 30 and large *n*_*j*_, all four estimators were unbiased. For *n*_*j*_ > 30, all four estimators were stable and bias decreased as *r* increased, with ${\hat{F}}_{st_{3}}$ the best estimator and ${\hat{F}}_{st_{1}}$ the worst estimator (Fig 4).

Application to Data: The means and variances of ${\hat{F}}_{st}$ values between the WETH and 11 samples in HapMap 3 are presented in Table 3. The WETH sample was closest to the MKK sample, consistent with shared Cushitic and Nilo-Saharan ancestry [13]. ${\hat{F}}_{st_{2}}$ and ${\hat{F}}_{st_{m}}$ yielded the same order for all pairs of relationships and all four estimators yielded the same order of relationships for the five HapMap samples closest to the WETH sample. The order of the means was ${\hat{F}}_{st_{1}}$ < ${\hat{F}}_{st_{m}}$ < ${\hat{F}}_{st_{2}}$ < ${\hat{F}}_{st_{3}}$. ${\hat{F}}_{st_{m}}$ was approximately 30% larger than ${\hat{F}}_{st_{1}}$ and approximately 20% smaller than ${\hat{F}}_{st_{2}}$, which has corresponding effects on divergence time estimates. Given that ${\hat{F}}_{st_{2}}$ and ${\hat{F}}_{st_{m}}$ are less downward biased than ${\hat{F}}_{st_{1}}$ for these sample sizes (Fig 2), the larger values are more likely to be correct.

**Table 3:** ${\hat{F}}_{st}$ between WETH and HapMap 3 samples.

	${\hat{F}}_{st_{1}}^{*}$	${\hat{F}}_{st_{2}}^{*}$	${\hat{F}}_{st_{m}}^{*}$	${\hat{F}}_{st_{3}}^{*}$
ASW	0.0155 (0.0005)	0.0222 (0.0016)	0.0185 (0.0008)	0.0226 (0.0016)
CEU	0.0368 (0.0023)	0.0630 (0.0067)	0.0499 (0.0042)	0.0632 (0.0067)
CHB	0.0624 (0.0059)	0.1012 (0.0139)	0.0815 (0.0094)	0.1044 (0.0146)
CHD	0.0629 (0.0059)	0.1021 (0.0141)	0.0822 (0.0095)	0.1052 (0.0147)
GIH	0.0359 (0.0022)	0.0603 (0.0063)	0.0479 (0.0039)	0.0611 (0.0064)
JPT	0.0634 (0.0060)	0.1029 (0.0142)	0.0828 (0.0096)	0.1060 (0.0149)
LWK	0.0210 (0.0008)	0.0343 (0.0023)	0.0276 (0.0014)	0.0351 (0.0024)
MKK	0.0081 (0.0002)	0.0121 (0.0005)	0.0101 (0.0003)	0.0122 (0.0005)
MXL	0.0371 (0.0023)	0.0601 (0.0067)	0.0474 (0.0039)	0.0612 (0.0067)
TSI	0.0344 (0.0020)	0.0578 (0.0058)	0.0459 (0.0036)	0.0586 (0.0060)
YRI	0.0264 (0.0011)	0.0451 (0.0035)	0.0358 (0.0021)	0.0454 (0.0036)

* Shown are means (variances) of ${\hat{F}}_{st}$.

## Discussion

*F*_*st*_ is directly related to the variance in allele frequencies among subpopulations. The dependence of *F*_*st*_ on allele frequencies and genetic diversity has been observed [14]. In our study, an approximately linear relationship between ${\hat{F}}_{st_{1}}$, ${\hat{F}}_{st_{2}}$, ${\hat{F}}_{st_{m}}$, and ${\hat{F}}_{st_{3}}$ with the squared difference of allele frequencies (${\hat{\vartheta }}^{2}$) was observed, as expected. By simulation, we found that all four estimators were unbiased for large numbers of subpopulations and individuals per subpopulation but that no one estimator was uniformly better than the others. For *F*_*st*_ < 0.5, ${\hat{F}}_{st_{1}}$ had smaller variances and MSE values. For *F*_*st*_ > 0.5, ${\hat{F}}_{st_{2}}$ had smaller variances and MSE values. For *F*_*st*_ ≈ 0.5, ${\hat{F}}_{st_{1}}$, ${\hat{F}}_{st_{2}}$, and ${\hat{F}}_{st_{m}}$ had similar variance and MSE values.

The numbers of individuals and markers have been reported to affect *F*_*st*_ estimation [5]. We found that the number of subpopulations was more important than the number of individuals per subpopulation. Estimation of *F*_*st*_, both in terms of means and variances, stabilized with approximately 30 subpopulations, regardless of the number of individuals per subpopulation. This behavior occurs because there are *r* estimates of ${\hat{p}}_{j}$ with which to estimate *p* and *σ* ^2^. Estimation was biased for *r* = 2 and improved as *r* increased, according to the Central Limit Theorem. Estimation was biased for *n*_*j*_ < 30 and improved as *n*_*j*_ increased (except for Wright’s estimator), also according to the Central Limit Theorem. Our proposed estimator is a minimum variance combination of Wright’s and Weir and Cockerham’s estimators and is less biased than Weir and Cockerham’s estimator for small samples sizes and less biased than Wright’s estimator for large sample sizes.

## Conclusion

A modified *F*_*st*_ estimator is proposed, which combines Wright’s and Weir and Cockerham’s estimators. It splits the difference in biases present in Wright’s and Weir and Cockerham’s estimators. We propose the routine use of this new and improved estimator of *F*_*st*_ as a way to reduce the biases and limitations of the classical estimators. We demonstrated that, in order to estimate *F*_*st*_ accurately, at least 30 subpopulations and 30 individuals per subpopulations are required.

## Appendix

### Proof of the Proposition

As $\bar{n}\to \infty$, $\bar{n}{\hat{F}}_{st_{1}}$ is asymptotically a chi-squared random variable, $S^{2}\overset{P}{\to }0$, $\bar{n}T_{1}∼\bar{n}S^{2}-\bar{p}(1-\bar{p})=\bar{p}(1-\bar{p})(\bar{n}{\hat{F}}_{st_{1}}-1)$, and

$$1+\frac{\left(r-1)(\bar{n}-n_{c}\right)}{\bar{n}-1}=\frac{\sum_{j=1}^{r}n_{j}^{2}/(r\bar{n})-1}{\bar{n}-1}=O(1).$$

Thus

$$T_{2}=[(n_{c}-1)/(\bar{n}-1)]\bar{p}(1-\bar{p})+O_{p}(1)$$

and

$$\bar{n}{\hat{F}}_{st_{2}}=\frac{\bar{n}-1}{n_{c}-1}(\bar{n}{\hat{F}}_{st_{1}}-1)+O_{p}(1).$$

Let

$$\delta =\underset{n\to \infty }{\text{lim}}\bar{n}/n_{c}$$

and $\sigma_{1}^{2}$ be the asymptotic variance of $\bar{n}{\hat{F}}_{st_{1}}$, then the asymptotic variance of $\bar{n}{\hat{F}}_{st_{2}}$ is $\delta^{2}\sigma^{2_{1}}$, and the asymptotic covariance of $\left(\bar{n}{\hat{F}}_{st_{1}},\bar{n}{\hat{F}}_{st_{2}}\right)$ is $\delta \sigma_{1}^{2}$. Now we have

$$\bar{n}{\hat{F}}_{st}(a)=[a+\delta (1-a)]\bar{n}{\hat{F}}_{st_{1}}-\delta (1-a)+O_{p}(1).$$

If *p*_1_ = ⋯ = *p*_2_, $\bar{p}(1-\bar{p})\overset{P}{\to }p_{0}(1-p_{0})$. Note the ${\hat{p}}_{j}$’s are independent, and

$$\bar{n}{\hat{F}}_{st_{1}}=\frac{1}{\left(r-1)p_{0}(1-p_{0}\right)}\sum_{j=1}^{r}n_{j}{\left(\hat{p_{j}}-\bar{p}\right)}^{2}+O_{p}(1).$$

Let $\hat{p}=({\hat{p}}_{1},\ldots ,{\hat{p}}_{r}{}^{)}\prime$, **p**_0_ = (*p*_0_, …, *p*_0_)’, *γ*_*j*_ = *n*_*j*_/*n*, and **b**_*j*_ = (−*γ*_1_, …, −*γ*_*j*−1_, (1 − *γ*_*j*_), −*γ*_*j*+1_, …, −*γ*_*r*_)′, then $b^{\prime }{}_{j}p_{0}=[(1-\gamma_{j})+\sum_{i\neq j}^{r}\gamma_{i}]p_{0}=0$ for *j* = 1, …, *r*, and so by the Central Limit Theorem,

$$\sqrt{n}({\hat{p}}_{j}-\bar{p})=\sqrt{n}b^{\prime }{}_{j}\hat{p}=\sqrt{n}b^{\prime }{}_{j}(\hat{p}-p_{0})\overset{D}{\to }N(0,\tau_{j}^{2}),$$

where $\tau_{j}^{2}=b^{\prime }{}_{j}\Omega b_{j}$, Ω = (*ω*_*ij*_)_*r*×*r*_ with *ω*_*ij*_ = *p*_0_(1 − *p*_0_) if *i* = *j* and *ω*_*ij*_ = 0 if *i* ≠ *j*.

Now we have, with $B=\sum_{j=1}^{r}\gamma_{j}b_{j}b^{\prime }{}_{j}$,

$$\begin{array}{c} \bar{n}{\hat{F}}_{st}(a)+\delta (1-a)=\frac{a+\alpha (1-a)}{\left(r-1)p_{0}(1-p_{0}\right)}\sum_{j=1}^{r}n_{j}{\left(\hat{p_{j}}-\bar{p}\right)}^{2}+O_{p}(1)\\ =\frac{a+\alpha (1-a)}{\left(r-1)p_{0}(1-p_{0}\right)}\sum_{j=1}^{r}\gamma_{j}nb^{\prime }{}_{j}(\hat{p}-p_{0}){\left(\hat{p}-p_{0}\right)}^{\prime }b_{j}+O_{p}(1)\\ =\frac{a+\alpha (1-a)}{\left(r-1)p_{0}(1-p_{0}\right)}n{\left(\hat{p}-p_{0}\right)}^{\prime }B(\hat{p}-p_{0})+O_{p}(1). \end{array}$$

Let Ω^−1/2^ be the square root of Ω: Ω = Ω′^1/2^Ω^1/2^, *λ*_1_, …, *λ*_*r*_ be all the eigenvalues of Ω′^1/2^ **B**Ω^1/2^, and Λ = *diag*(*λ*_1_, …, *λ*_*r*_), then there is an orthogonal normal matrix **Q** such that Ω′^1/2^ **B**Ω^1/2^ = **Q**′Λ**Q**, and so

$$n{\left(\hat{p}-p_{0}\right)}^{\prime }B(\hat{p}-p_{0})\overset{D}{\to }\sum_{j=1}^{r}\lambda_{j}\chi_{j}^{2},$$

where the $\chi_{j}^{2}$’s are independent chi-squared random variables with one degree of freedom. This gives the desired result.

Lastly, we prove

$$\delta =\underset{n\to \infty }{\text{lim}}\frac{\bar{n}}{n_{c}}\geq 1,\;\text{with}\;“=”\;\text{if and only if}\;n_{1}=\cdots =n_{r}.$$

In fact,

$$(r-1)(\bar{n}-n_{c})=\frac{\sum_{j=1}^{r}n_{j}^{2}}{\sum_{j=1}n_{j}}-\frac{1}{r}\sum_{j=1}^{r}n_{j}=\frac{r\sum_{j=1}^{r}n_{j}^{2}-{\left(\sum_{j=1}^{r}n_{j}\right)}^{2}}{r\sum_{j=1}^{r}n_{j}}.$$

It is known that for *r* = 1 or 2, $r\sum_{j=1}^{r}n_{j}^{2}-{\left(\sum_{j=1}^{r}n_{j}\right)}^{2}\geq 0$ with “=” if and only if *n*_1_ = ⋯ = *n*_*r*_. Now we use induction to prove this is true for all integer *r*. In fact, suppose the above conclusion is true for some integer *r* > 2, then for integer *r* + 1,

$$A_{n}:=(r+1)\sum_{j=1}^{r+1}n_{j}^{2}=r\sum_{j=1}^{r}n_{j}^{2}+\sum_{j=1}^{r}n_{j}^{2}+(r+1)n_{r+1}^{2},$$

and

$$B_{n}:={\left(\sum_{j=1}^{r+1}n_{j}\right)}^{2}={\left(\sum_{j=1}^{r}n_{j}\right)}^{2}+2n_{r+1}\sum_{j=1}^{r}n_{j}+n_{r+1}^{2}.$$

Since by assumption $r\sum_{j=1}^{r}n_{j}^{2}\geq {\left(\sum_{j=1}^{r}n_{j}\right)}^{2}$,

$$\begin{array}{l} A_{n}-B_{n}\geq \sum_{j=1}^{r}n_{j}^{2}+(r+1)n_{r+1}^{2}-2n_{r+1}\sum_{j=1}^{r}n_{j}-n_{r+1}^{2}\\ =\sum_{j=1}^{r}n_{j}^{2}+rn_{r+1}^{2}-2n_{r+1}\sum_{j=1}^{r}n_{j}=\sum_{j=1}^{r}(n_{j}^{2}+n_{r+1}^{2}-2n_{j}n_{r+1})\geq 0 \end{array}$$

with “=” if and only if *n*_1_ = ⋯*n*_*r*+1_, since $n_{j}^{2}+n_{r+1}^{2}-2n_{j}n_{r+1}={\left(n_{j}-n_{r+1}\right)}^{2}\geq 0$, with “=” if and only if *n*_*j*_ = *n*_*r*+1_.

This gives *δ* ≥ 1 with “=” if and only if *n*_1_ = ⋯ = *n*_*r*_.

## Supporting Information

S1 TableMeans, Variances, and MSEs of ${\hat{F}}_{st}$ in simulation 2.(PDF)
