# Supplementary material for: An Improved Fst Estimator
Source: PLoS One. 2015 Aug 28;10(8):e0135368. doi: 10.1371/journal.pone.0135368 (PMC4552798; doi:10.1371/journal.pone.0135368)
Supplement: S1 Table — (PDF) [file pone.0135368.s001.pdf]

S1 Table. Means, Variances, and MSEs of $\hat{F}_{st}$ in simulation 2

p	True F_{st}	$\hat{F}^{*}_{st_1}$	$\hat{F}_{st_2}$	$\hat{F}_{st_m}$	$\hat{F}_{st_3}$
0.1	0.1	9.93E-02, 1.40E-04, 1.40E-04	9.93E-02, 1.41E-04, 1.41E-04	9.93E-02, 1.40E-04, 1.41E-04	9.98E-02, 1.41E-04, 1.41E-04
	0.2	1.99E-01, 5.72E-04, 5.73E-04	1.99E-01, 5.76E-04, 5.77E-04	1.99E-01, 5.74E-04, 5.75E-04	1.99E-01, 5.75E-04, 5.75E-04
	0.3	2.97E-01, 1.15E-03, 1.16E-03	2.98E-01, 1.15E-03, 1.16E-03	2.97E-01, 1.15E-03, 1.16E-03	2.98E-01, 1.15E-03, 1.16E-03
	0.4	3.97E-01, 1.78E-03, 1.79E-03	3.98E-01, 1.78E-03, 1.79E-03	3.97E-01, 1.78E-03, 1.79E-03	3.98E-01, 1.78E-03, 1.78E-03
	0.5	4.96E-01, 2.20E-03, 2.22E-03	4.97E-01, 2.20E-03, 2.21E-03	4.96E-01, 2.20E-03, 2.21E-03	4.97E-01, 2.20E-03, 2.21E-03
	0.6	5.96E-01, 2.26E-03, 2.28E-03	5.97E-01, 2.26E-03, 2.27E-03	5.96E-01, 2.26E-03, 2.28E-03	5.97E-01, 2.26E-03, 2.27E-03
	0.7	6.96E-01, 2.22E-03, 2.24E-03	6.97E-01, 2.21E-03, 2.22E-03	6.96E-01, 2.22E-03, 2.23E-03	6.97E-01, 2.21E-03, 2.22E-03
	0.8	7.97E-01, 1.70E-03, 1.71E-03	7.98E-01, 1.69E-03, 1.69E-03	7.98E-01, 1.70E-03, 1.70E-03	7.98E-01, 1.69E-03, 1.69E-03
	0.9	8.98E-01, 9.71E-04, 9.74E-04	8.99E-01, 9.65E-04, 9.66E-04	8.98E-01, 9.68E-04, 9.70E-04	8.99E-01, 9.64E-04, 9.65E-04
0.3	0.1	9.97E-02, 8.11E-05, 8.12E-05	9.97E-02, 8.18E-05, 8.19E-05	9.97E-02, 8.15E-05, 8.16E-05	1.00E-01, 8.18E-05, 8.18E-05
	0.2	1.99E-01, 2.68E-04, 2.69E-04	1.99E-01, 2.70E-04, 2.70E-04	1.99E-01, 2.69E-04, 2.69E-04	2.00E-01, 2.69E-04, 2.69E-04
	0.3	2.99E-01, 4.67E-04, 4.68E-04	3.00E-01, 4.70E-04, 4.69E-04	3.00E-01, 4.68E-04, 4.68E-04	3.00E-01, 4.69E-04, 4.69E-04
	0.4	3.99E-01, 6.62E-04, 6.64E-04	4.00E-01, 6.64E-04, 6.64E-04	3.99E-01, 6.63E-04, 6.64E-04	4.00E-01, 6.64E-04, 6.64E-04
	0.5	4.99E-01, 7.86E-04, 7.88E-04	5.00E-01, 7.87E-04, 7.87E-04	4.99E-01, 7.87E-04, 7.88E-04	5.00E-01, 7.86E-04, 7.86E-04
	0.6	5.98E-01, 8.34E-04, 8.36E-04	5.99E-01, 8.33E-04, 8.33E-04	5.99E-01, 8.34E-04, 8.35E-04	6.00E-01, 8.32E-04, 8.32E-04
	0.7	6.99E-01, 7.68E-04, 7.70E-04	6.99E-01, 7.66E-04, 7.66E-04	6.99E-01, 7.67E-04, 7.68E-04	7.00E-01, 7.65E-04, 7.65E-04
	0.8	7.99E-01, 6.24E-04, 6.25E-04	8.00E-01, 6.21E-04, 6.21E-04	7.99E-01, 6.23E-04, 6.23E-04	8.00E-01, 6.21E-04, 6.21E-04
	0.9	8.99E-01, 3.63E-04, 3.63E-04	9.00E-01, 3.60E-04, 3.60E-04	8.99E-01, 3.61E-04, 3.62E-04	9.00E-01, 3.60E-04, 3.60E-04
0.4	0.1	9.96E-02, 7.88E-05, 7.90E-05	9.96E-02, 7.95E-05, 7.97E-05	9.96E-02, 7.92E-05, 7.93E-05	1.00E-01, 7.94E-05, 7.94E-05
	0.2	1.99E-01, 2.40E-04, 2.41E-04	2.00E-01, 2.42E-04, 2.42E-04	1.99E-01, 2.41E-04, 2.41E-04	2.00E-01, 2.42E-04, 2.42E-04
	0.3	2.99E-01, 4.18E-04, 4.20E-04	2.99E-01, 4.21E-04, 4.21E-04	2.99E-01, 4.19E-04, 4.20E-04	3.00E-01, 4.20E-04, 4.20E-04
	0.4	3.99E-01, 5.62E-04, 5.63E-04	4.00E-01, 5.64E-04, 5.64E-04	3.99E-01, 5.63E-04, 5.63E-04	4.00E-01, 5.63E-04, 5.63E-04
	0.5	4.99E-01, 6.63E-04, 6.64E-04	5.00E-01, 6.63E-04, 6.63E-04	4.99E-01, 6.63E-04, 6.64E-04	5.00E-01, 6.63E-04, 6.63E-04
	0.6	5.99E-01, 7.01E-04, 7.03E-04	6.00E-01, 7.00E-04, 7.01E-04	5.99E-01, 7.01E-04, 7.02E-04	6.00E-01, 7.00E-04, 7.00E-04
	0.7	6.99E-01, 6.50E-04, 6.52E-04	6.99E-01, 6.48E-04, 6.48E-04	6.99E-01, 6.49E-04, 6.50E-04	7.00E-01, 6.48E-04, 6.48E-04
	0.8	7.99E-01, 5.26E-04, 5.27E-04	8.00E-01, 5.23E-04, 5.23E-04	7.99E-01, 5.25E-04, 5.25E-04	8.00E-01, 5.23E-04, 5.23E-04
	0.9	9.00E-01, 3.09E-04, 3.10E-04	9.00E-01, 3.07E-04, 3.07E-04	9.00E-01, 3.08E-04, 3.08E-04	9.00E-01, 3.07E-04, 3.07E-04
0.5	0.1	9.94E-02, 7.44E-05, 7.48E-05	9.94E-02, 7.51E-05, 7.55E-05	9.94E-02, 7.48E-05, 7.51E-05	9.98E-02, 7.50E-05, 7.50E-05
	0.2	1.99E-01, 2.31E-04, 2.32E-04	2.00E-01, 2.33E-04, 2.33E-04	1.99E-01, 2.32E-04, 2.32E-04	2.00E-01, 2.33E-04, 2.33E-04
	0.3	2.99E-01, 3.93E-04, 3.95E-04	3.00E-01, 3.95E-04, 3.95E-04	2.99E-01, 3.94E-04, 3.95E-04	3.00E-01, 3.95E-04, 3.95E-04
	0.4	3.99E-01, 5.44E-04, 5.44E-04	4.00E-01, 5.45E-04, 5.45E-04	4.00E-01, 5.44E-04, 5.45E-04	4.00E-01, 5.45E-04, 5.45E-04
	0.5	4.99E-01, 6.17E-04, 6.18E-04	5.00E-01, 6.18E-04, 6.18E-04	4.99E-01, 6.17E-04, 6.17E-04	5.00E-01, 6.17E-04, 6.17E-04
	0.6	5.99E-01, 6.51E-04, 6.52E-04	6.00E-01, 6.50E-04, 6.50E-04	6.00E-01, 6.50E-04, 6.51E-04	6.00E-01, 6.50E-04, 6.50E-04
	0.7	6.99E-01, 6.21E-04, 6.23E-04	7.00E-01, 6.20E-04, 6.20E-04	6.99E-01, 6.20E-04, 6.21E-04	7.00E-01, 6.19E-04, 6.19E-04
	0.8	7.99E-01, 4.89E-04, 4.89E-04	8.00E-01, 4.86E-04, 4.86E-04	7.99E-01, 4.87E-04, 4.88E-04	8.00E-01, 4.86E-04, 4.86E-04
	0.9	9.00E-01, 2.94E-04, 2.95E-04	9.00E-01, 2.92E-04, 2.92E-04	9.00E-01, 2.93E-04, 2.93E-04	9.00E-01, 2.92E-04, 2.92E-04

[*] Means, variances, and Mean Squared Errors (MSEs) from 200 subpopulations with 1000 individuals per subpopulation, given p and F_{st}.
